# Characterization of Bromelain‐Soluble Sheepskin (*Ovis aries*) Proteins and Effect of In Vitro Gastrointestinal Digestion on Angiotensin Converting Enzyme Inhibition Activity

**DOI:** 10.1002/fsn3.71396

**Published:** 2026-01-28

**Authors:** Dita Prameswari Trenggono Putri, Marta Gallego, Mohammad Zainal Abidin, Nanung Agus Fitriyanto, Leticia Mora, Fidel Toldrá, Yuny Erwanto

**Affiliations:** ^1^ Instituto de Agroquímica y Tecnología de Alimentos (CSIC) Paterna Valencia Spain; ^2^ Department of Animal Products Technology, Faculty of Animal Science Gadjah Mada University Yogyakarta Indonesia

**Keywords:** ACE‐inhibition, bioactive peptides, in silico analysis, in vitro digestion, proteins, sheepskin

## Abstract

This work characterizes proteins extracted from sheepskin using bromelain and examines angiotensin converting enzyme (ACE) inhibition activity of the peptides generated by in vitro gastrointestinal digestion (GID). Gel electrophoresis revealed three major protein bands corresponding to keratin and collagen. In vitro gastrointestinal digestion facilitated protein breakdown into peptides, yielding below 3 kDa of molecular weight peptides. Arginine, tyrosine, lysine, and leucine were identified as the major residues of amino acids in the bromelain‐soluble protein hydrolysate (BSPH). The action of inhibiting ACE of 1 mg/mL of BSPH was 48.99%, whereas the activity of the < 3 kDa ultra‐filtrated fractions obtained before and after in vitro GID were 0% and 19.94%, respectively. To acquire the peptide profile and fractionate the ultra‐filtrated peptide fractions, reverse‐phase high‐performance liquid chromatography (RP‐HPLC) was utilized. Among all the RP‐HPLC fractions, 2, 3, 12, and 15 had the most active ACE inhibition. Subsequently, the peptide sequences from four active fractions were recognized using tandem mass spectrometry (MS/MS) and followed by in silico analysis to examine their bioactivity. Two potent ACE‐inhibitory peptide candidates from sheepskin collagen, GPAGPAGPR, and QGPPGPAGPR, were identified by in silico analysis.

## Graphical abstract

Novel bioactive peptides resulted from in vitro gastrointestinal digestion of soluble sheep skin protein.

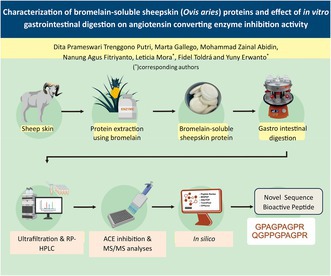

## 1 Introduction

Animal by‐products have been recognized as a sustainable protein supply, containing peptides that possess biological bioactivity. Sheepskin is a non‐edible by‐product produced by slaughterhouses with a high content of proteins. The valorization of this by‐product would provide added value and, at the same time, reduce the problems associated with waste disposal. Skin proteins can be acquired through enzymatic extraction, utilizing papain, pepsin, bromelain, and trypsin. Proteases are frequently employed in protein extraction using the dilute acetic acid method, so that the enzymatic hydrolysis of some structural protein matrices produces a greater yield of protein extraction compared to the method without enzyme. Commercial proteases derived from animals are more difficult to obtain than plant or microorganism‐based enzymes because farming and slaughtering processes are time‐consuming and expensive. In this regard, plant‐derived proteases such as bromelain can be offered as an alternative enzyme to extract skin proteins and generate bioactive peptides, producing a high protein yield compared to other proteases like pepsin (Devita et al. 2021).

Bromelains are cysteine peptidases with strong proteolytic activity, specifically endopeptidase action, and can exert their activity efficiently across a wide range of pH levels and temperatures, which makes them versatile in different food applications. This enzyme is also relatively cost‐effective and eco‐friendly, particularly when obtained from pineapple waste. Stem bromelain is known for its cleavage specificity at benzyloxycarbonyl‐Arg‐Arg, which results in peptides enriched in hydrophobic and arginine residues. These sequence characteristics have been demonstrated to enhance the bioactive potential produced by peptides (Tacias‐Pascacio et al. 2023).

Generally, bioactive peptides have between two and twenty amino acid residues that can exhibit a physiological effect in the organism besides their nutritional properties. Diverse biological activities are exhibited by bioactive peptides, such as antihypertensive, antidiabetic, antimicrobial, antioxidant, antiviral, immunomodulatory, cytomodulatory, hypocholesterolemic, wound‐healing, and mineral absorption–enhancing effects (Xiao et al. 2022). Among the various bioactivities, ACE‐inhibitory activity is especially noteworthy because of its potential link to antihypertensive properties. Hypertension has been recognized as the primary contributor to premature mortality worldwide (World Health Organization (WHO) 2023). The ACE plays a crucial part in the renin–angiotensin system (RAS) because of its responsibility for transforming angiotensin I into angiotensin II, which can lead to the rise of blood pressure in the human body. Angiotensin II, as the primary effector of the RAS, regulates systemic arterial pressure through vasoconstriction, modulation of sympathetic nervous system activity, as well as stimulation of renal salt and water retention. Accordingly, inhibition of ACE has become the basis for a widely used class of antihypertensive medications (Yang et al. 2021).

Since synthetic ACE inhibitors have undesirable side effects, bioactive peptides obtained from animal by‐products could be considered an interesting option for obtaining ACE inhibitors. Fan et al. (2020) indicate that peptides interact with residues in the active site of ACE, which leads to a reduction in ACE enzymatic activity. Animal skin protein contains a high amount of collagen, which is a protein rich in proline residues that has a strong potential to generate peptides with ACE‐inhibitory activity. Collagen‐derived peptides have been widely reported as effective natural ACE inhibitors due to their specific amino acid composition, particularly the presence of proline, which enhances their binding affinity to the ACE active site and contributes to antihypertensive effects (Hou et al. 2023).

Bioactive peptides were generated from the protein through enzymatic hydrolysis using proteases. Numerous proteases have been utilized in prior studies for protein hydrolysis, such as alcalase, bromelain, flavourzyme, papain (López‐Pedrouso et al. 2020), and protana prime (Ozturk‐Kerimoglu et al. 2023). Accordingly, many previous studies primarily focused on protein hydrolysis utilizing several random enzyme combinations to generate bioactive peptides, without specifically considering physiological digestion mechanisms. In contrast, the present study emphasizes bioactive peptides produced through a simulated gastrointestinal digestion (GID) process, which closely mimics the sequential enzymatic actions occurring in the human digestive tract. Several studies have used gastrointestinal digestion (GID) to release bioactive peptides from protein, utilizing pepsin and pancreatin enzymes from different sources of animal proteins, such as milk (He et al. 2024), fish collagen (Liang et al. 2014), and goat meat (Luasiri et al. 2024). However, bioactive peptides generated from Indonesian local sheepskin through a combination of bromelain extraction followed by GID have not been explored yet. This study lies in the identification of novel ACE‐inhibitory peptides from the skin of the Garut sheep, a local sheep breed that is primarily raised in West Java, Indonesia. This work represents the first systematic investigation of bioactive peptides from a livestock source that is endemic to Indonesia, filling a geographic and species‐specific gap in the peptide literature. Therefore, the objective of this research was to recognize novel natural angiotensin‐converting enzyme inhibition peptides derived from Indonesian local sheepskin proteins, obtained through bromelain‐assisted extraction followed by gastrointestinal digestion utilizing pepsin and pancreatin enzymes.

## 2 Materials and Methods

### 2.1 Reagents and Chemicals

Bromelain from pineapple stem (3 U/mg), rabbit lung angiotensin I‐converting enzyme (ACE), porcine bile extract, pancreatic pancreatin, gastric mucosal pepsin, captopril, DL‐Dithiothreitol (DTT), o‐phthaldialdehyde (OPA), trifluoroacetic acid (TFA), serine, sodium dodecyl sulfate (SDS), and formic acid (FA) were from Sigma Aldrich Co., manufactured in St. Louis, MO, USA. The substrate Abz‐Gly‐Phe (NO_2_)‐Pro trifluoroacetate salt was obtained from Bachem A. G., manufactured in Bubendorf, Switzerland. Acetonitrile (ACN) multi solvent HPLC grade was obtained from Scharlab, S.L., manufactured in Barcelona, Spain. Calcium chloride and di‐sodium tetraborate 10‐hydrate were obtained from the brand Panreac Química, S.A.U., manufactured in Barcelona, Spain.

### 2.2 Materials

A male 18‐month‐old Indonesian Garut Sheepskin ( *Ovis aries* ) purchased from a local market in Cirebon, West Java, Indonesia, was used in this research. Hair located on the skin was removed using a hair shaver and a cutter knife. A fleshing machine was used to remove the rest of the meat and fat that was still attached to the skin. Once cleaned, the skin was sliced into little fragments of around 100 g each and preserved at −20°C till subsequent utilization.

### 2.3 Methods

#### 2.3.1 Extraction of Sheepskin Proteins

The procedure for extracting proteins was carried out with the published collagen extraction technique by Hakim et al. (2021), with slight adjustments, considering that collagen is the predominant protein in livestock skin (Kotowicz et al. 2024; Matinong et al. 2022). A 100 g of sheepskin was thawed under room temperature conditions for 1 h and immersed in Sodium hydroxide (NaOH) at a concentration of 0.1 M with a skin and solvent ratio of 1:10 (w/v). The soaking process was conducted for half an hour with continuous stirring. After that, the protein extraction process was conducted by soaking the skin in 0.5 M acetic acid, pH 4.5, using a ratio of 1:10 (w/v) containing 0.05% (w/v) bromelain enzyme (1.50 U/mL), then incubating it for 24 h in a refrigerator. This extraction conditions are generally optimized to preserve the native structure and solubility of collagen rather than to achieve maximal proteolytic activity of the enzyme (Munmun et al. 2024) to retain its collagenous characteristics, which are crucial for subsequent hydrolysis and ACE inhibitory bioactive peptide generation.

The extract was filtered, and proteins were precipitated by the addition of 2.6 M NaCl, with stirring until dissolved, then stored in the refrigerator overnight. Afterward, centrifugation was performed at 10,000 ***g*** speed for 1 h in the refrigerator. The resultant dry matter was re‐dissolved in 0.02 M acetic acid and subjected to dialysis process using cellulose dialysis tubing with a molecular weight cut‐off (MWCO) of 14 kDa, by brand Sigma Aldrich, manufactured in St. Louis, MO, USA. The dialysis process was conducted in a refrigerator for 48 h, with buffer changes every 3 h. The bromelain‐soluble sheepskin protein (BSP) extract was subsequently obtained by freeze‐drying. All procedures were performed in quintuplicate. The yield of the sheepskin protein extraction was determined using the formula that follows:

(1) $$\text{Collagen yields}\;\left(\%\right)=\left(\mathrm{Wet}\;\text{weight of collagen}/\mathrm{Wet}\;\text{weight of material used}\right)\times 100\%$$

#### 2.3.2 Protein Content Assessment

The dumas combustion method was used to assess the protein content of BSP. The analysis was performed using a Rapid N Exceed nitrogen analyzer (Elementar analyses system GmbH, Langenselbold, Germany). The methods were conducted in accordance with AOAC method 992.15 (AOAC 1992).

#### 2.3.3 Simulated Gastrointestinal Digestion (GID)

Bromelain‐soluble protein (BSP) was digested in vitro in accordance with the standardized INFOGEST protocol for gastrointestinal digestion (GID) reported by Minekus et al. (2014) and Brodkorb et al. (2019). Before the digestion process was conducted, the enzymatic activity and the bile concentration were assessed. For digestion, in brief, 20 mg of BSP were solubilized in 1 mL of 0.01 M hydrochloric acid and combined with 1 mL of pH 3 simulated gastric fluid containing pepsin at a concentration of 2000 U/mL. For 2 h, the mixture was incubated at 37°C while gently stirring. Subsequently, 2 mL of pH 7 simulated intestinal fluid containing bile with a concentration of 10 mmol/L and pancreatin of 100 U trypsin activity/mL was added to the mixed solution. After that, the mixed solution was incubated again for 2 h at 37°C. To stop the digestion, the solutions were heat shocked by heating for 5 min at 95°C and quickly chilled on ice for 10 min. The hydrolysate was then centrifuged with centrifugal speed at 8000 ***g*** for 10 min, and the supernatants of bromelain‐soluble protein hydrolysates (BSPH) were collected for subsequent analyses.

#### 2.3.4 Protein Molecular Weight Analysis Using SDS‐PAGE

The molecular weight profile of BSP and BSPH was measured by SDS‐PAGE analysis using the Laemmli technique (Laemmli 1970) with slight adjustment. Samples collected before and after GID were diluted to 1 mg/mL in 1× Laemmli sample buffer. The solution was then denatured by heat shock at 95°C in the water bath for 5 min. Twenty‐five microliters of each sample were placed onto a 12% Bio‐Rad gel, and electrophoresis of the sample was conducted for 30 min at 200 V. Following the electrophoresis process, the gel was set by soaking in 40% ethanol or 10% acetic acid for 1 h. The fixed gel was then colored with colloidal Coomassie from Bio‐Rad Laboratories, manufactured in Madrid, Spain, for 1 h, and then destained with bidistilled water to get the clear protein band profile. The Image Scanner (GE Healthcare) was used to obtain the gel images, and protein band profiles in the gel images were processed by ImageJ software v.1.52 made by Wayne Rasband, National Institute of Mental Health, created in the USA.

#### 2.3.5 Mass Spectrometry Identification of Protein Bands From SDS‐PAGE

Protein identification in BSP was performed by in‐gel digestion of the ten main SDS‐PAGE bands (Figure S1) using trypsin for sequencing‐grade at a concentration of 0.5 ng (Promega, Fitchburg, WI, USA), following the procedure of Shevchenko et al. (1996). The tryptic hydrolysis was terminated using 10% trifluoroacetic acid. Subsequently, 4 μL of the digest was dissolved to 20 μL FA (0.1%). The mixture was placed on an Evotip Pure tip from Evosep in accordance with the manufacturer's guidelines.

Peptide separation and analysis were carried out using an EvoSep One liquid chromatography system combined with a timsTOF fleX mass spectrometer from Bruker Daltonik GmbH, manufactured in Bremen, Germany. Peptides were eluted on a PepSep C18 analytical column with specification 15 cm × 150 μm, 1.5 μm from Evosep with the manufacturer‐defined 30 SPD gradient. Ionization was achieved with a Captive Spray source at 200°C and 1700 V, and spectra were obtained in positive polarity over an m/z scale of 100–1700. The parameters for the custom trapped ion mobility spectrometry (TIMS) were 1/K0 0.6–1.4 V·s/cm^2^, ramp period of 100 ms, and ramp rate of 9.42 Hz. Data acquisition was executed in DDA‐PASEF mode with a total cycle duration of half a second and four PASEF ramps. With active exclusion turned on, an intensity threshold of 1000 and a goal intensity of 12,500 have been used. For protein identification, MS Fragger via the Frag Pipe platform was employed to find the UniProt Caprinae database with trypsin specificity. Normalized spectral abundance factors (NSAF), calculated from spectral counting, were used to assess relative protein abundance.

#### 2.3.6 Degree of Hydrolysis Measurement of the Hydrolysate

The percentage DH value of hydrolysate was assessed by the o‐phthaldialdehyde (OPA) analysis as outlined in Nielsen et al. (2001), with slight changes. Before conducting the analysis, the OPA solution was made from several chemicals. In brief, the first solution was made by dissolving 200 mg SDS and 7.62 g di‐Natetraborate decahydrate in 150 mL of bidistilled water. Separately, 160 mg of o‐phthaldialdehyde reagent was diluted in four milliliters ethanol then mixed with the previous solution. Additionally, 176 mg DTT had been included in the mixture, then bidistilled water was added to the mixture until it reached a final volume. For the assay, 36 μL of blank (bidistilled water), serine standard, or sample (BSPH) was combined with OPA reagent at 270 μL in the microplate. Following that, the combined solution was placed in a CLARIOstar Plus multimode microplate from BMG LABTECH GmbH, manufactured in Ortenberg, Germany, and reacted for 2 min at 340 nm absorbance.

The value of DH (%) was calculated according to the Equation (2):

(2) $$\mathrm{DH}\;\left(\%\right)=h/\text{htot}\times 100$$

where h signifies the quantity of hydrolyzed peptide bonds, htot indicates the total number of peptide bonds per protein equivalent. The value of htot was determined from the gelatin data provided by Adler‐Nissen (1986).

#### 2.3.7 Analysis of Free Amino Acid

An Acquity ArcTM UHPLC system by Waters, Milford, MA, manufactured in the USA, was used to assess the free amino acid (aa) content of BSP, BSPH, and control. Briefly, 200 μL of samples with a 5 mg/mL concentration were deproteinized using fifty microliters of the 10 mM of α‐aminobutyric acid and 750 μL acetonitrile (ACN). The solution underwent centrifugation at 20,000 ***g*** speed for 5 min, after which the supernatant was derivatized utilizing the AccQ‐Tag Ultra derivatization kit (Waters) in accordance with the manufacturer's guidelines. In the derivatization process, 10 μL of calibration standard or deproteinized sample was combined with twenty microliters derivatization reagent and seventy microliters borate buffer, incubated at ambient temperature for 60 s, and then heated at 55°C for 10 min.

A 2 μL amount of the samples was injected onto an AccQ‐Tag Ultra C18 column with specification 2.5 μm, 4.6 × 150 mm, from Waters, and maintained at 43°C, using a 1.5 mL/min flow rate. Separation was achieved using a four‐solvent system (eluent A was 100% AccQ‐Tag Ultra eluent A; eluent B was 10% eluent B in bidistilled water; eluent C was water; eluent D was 100% eluent B) under a 36‐min gradient program as recommended by the manufacturer. Detection was performed with a PDA detector at 260 nm, and data collection and analysis were carried out using Empower 3 software (Waters). Each analysis was conducted in triplicate.

#### 2.3.8 Assessment of ACE Inhibition Activity

The efficacy of ACE inhibition was evaluated with the fluorescence‐based technique established by Sentandreu and Toldrá (2006). In summary, 50 μL samples were put in the microplate, which was followed by 50 μL ACE solution (3 mU/mL). The ACE solution was previously prepared by diluting ACE in a pH 8.3 Tris‐base buffer at a concentration of 150 mM. The reaction commenced by the addition of two hundred 0.45 mM Abz‐Gly‐Phe‐(NO_2_)‐Pro in buffer as a substrate solution. The substrate solution was previously made by dissolving Abz‐Gly‐Phe‐(NO₂)‐Pro in a pH 8.3 Tris–HCl buffer containing 1.125 M NaCl, with a buffer concentration of 150 mM. The mixture was immediately incubated in a CLARIOstar Plus multimode microplate for 1 h at 37°C, with fluorescence absorbance determined at 355 nm for excitation wavelength and 405 nm for emission wavelength. Captopril was utilized as a positive control, whereas the bidistilled water is the negative control. Three duplicates of each measurement were made, and the % inhibition of ACE‐inhibitory activity was shown.

#### 2.3.9 Ultrafiltration (UF) Separation

Bromelain‐soluble sheepskin protein and BSPH samples (500 μL, 50 mg/mL) were separated by size using serial ultrafiltration with Amicon Ultra 0.5 mL centrifugal filters from Merck Millipore Ltd., manufactured in Cork, Ireland. The MWCO of the filters was 30, 10, and 3 kDa, resulting in four fractions, including > 30, 10–30, 3–10, and < 3 kDa, that were lyophilized and weighed to ascertain the peptide molecular distribution data.

#### 2.3.10 Peptides Profiling and Fractionation by RP‐HPLC

Profiling and fractionation of peptides from the selected UF fraction were conducted using RP‐HPLC, Agilent 1100 HPLC by Agilent Technologies, manufactured in Palo Alto, CA, USA, equipped with a Symmetry C18 column (5 μm, 4.6 × 250 mm) by Waters Co., Milford, MA, manufactured in the USA. The mobile phases consisted of trifluoroacetic acid at a concentration of 0.1% (phase A) using water solvent, and trifluoroacetic acid at a concentration of 0.085% with a ratio of acetonitrile: water of 60:40, v/v (phase B). A gradient program comprising 2% phase B for 5 min and a linear increase to 45% phase B over 50 min was used to inject 10 μL of material and elute it using a 1 mL/min flow rate for peptide profiling. Elution was observed at 214 nm of ultraviolet absorption, and the profile was collected. For peptide fractionation, in brief, 100 μL of the fraction sample was then injected under similar chromatographic conditions for 50 min and the fraction was collected every 2 min, resulting in a total of 25 tubes, each containing 2 mL. Tandem mass spectrometry analysis was then used to identify ACE‐inhibitory activity.

#### 2.3.11 Identification of Peptides With LC–MS/MS

The tandem mass spectrometry (MS/MS) system outlined in Section 2.3.4 was applied to determine the peptide sequences that were present in the RP‐HPLC fractions that were chosen for the most active. Briefly, 2 μL of the selected RP‐HPLC fraction sample was inserted onto an EvoTip Pure tip, and peptides were subsequently placed onto a PepSep analytical column using a pre‐set 60 SPD chromatographic technique, which employed a 21 min with 1 μL/min gradient flow rate. The mobile phases consisted of phase A and phase B, where phase A consisted of 0.1% formic acid diluted in water, and phase B consisted of 0.1% formic acid dissolved in acetonitrile. The ionization parameters, spectral acquisition, and TIMS settings were applied as described in Section 2.3.3. Peak identification and database queries were conducted using Mascot Distiller v2.7.1 from Matrix Science Inc., created in Boston, MA, USA, with the link http://www.matrixscience.com against the UniProt database, which is categorized under the Chordata taxonomy. The criteria for the search were created without any specificity for the enzyme. The peptides' mass tolerance was set at 100 ppm, and the tolerance for tandem mass spectrometry was set at 0.3 da. The *p* < 0.05 was set for significance threshold.

#### 2.3.12 In Silico Analysis of ACE Inhibitory Peptides Using Bioinformatics Tools

The bioactivity prediction of all discovered peptides was assessed by *in silico*, utilizing several bioinformatic tools. Peptide Ranker was applied with the link http://bioware.ucd.ie/~compass/biowareweb/ to calculate peptide scores on a scale from 0 to 1, with values nearer to 1 signaling a high probability of bioactivity. Peptides showing a Peptide Ranker Ratio (PRR) over 0.85 were selected for further analysis. Peptides were characterized as prospective bioactive precursors utilizing the BIOPEP database with the link http://www.uwm.edu.pl/biochemia/index.php/en/biopep, which estimates the frequency of bioactive fragment occurrence (A) in a sequence according to published data. Potential allergenicity prediction was assessed using AllerTOP with the link http://www.ddg‐pharmfac.net/AllerTOP/index.html. AllerTOP can classify detected peptides as possible allergens or non‐allergens according to their physicochemical attributes. Peptide toxicity and physicochemical properties were assessed using ToxinPred with the link http://crdd.osdd.net/raghava/toxinpred/ through an SVM‐based technique. ToxinPred uses a threshold of 0.0 to differentiate between peptides that are toxic and those that are not toxic. Lastly, the prediction of cell‐penetrating potential peptides was assessed using CPPpred with the link http://distilldeep.ucd.ie/CPPpred/, which estimates the likelihood of peptides to cross cell membranes and enter the bloodstream.

#### 2.3.13 Statistical Analysis of the Study

The statistical studies were analyzed using one‐way analysis of variance (ANOVA), and significant differences among means were further investigated using Fisher's Least Significant Difference (LSD) test utilizing OriginPro version 2023b from Origin Lab Corporation, created in Northampton, MA, USA. The findings are presented in the form of the mean and the standard deviation (SD) of the replicates collected. A *p* < 0.05 significance threshold is used to evaluate the differences between replicates. Moreover, Microsoft Excel was employed for graph generation.

## 3 Results and Discussion

### 3.1 Effect of Gastrointestinal Digestion (In Vitro) on Bromelain‐Soluble Sheepskin Proteins

The yield of bromelain‐soluble sheepskin protein extract (BSP) extraction was 66.64%, with protein content reaching 55%. Subsequently, the BSP was subjected to simulated gastrointestinal digestion, resulting in bromelain‐soluble sheepskin protein hydrolysate (BSPH). Both BSP and BSPH were characterized using SDS‐PAGE, and the corresponding BSP protein of SDS‐PAGE was recognized by the LC–MS/MS analysis system and presented in Figure S1. The electrophoresis pattern of BSP showed nine protein bands with major molecular weights of around 140, 100, and 10 kDa, whereas BSPH remained a minor protein, around 50–10 kDa, after GID. Ten abundant proteins were then recognized using LC–MS/MS, corresponding to keratin, collagen, and others, as displayed in Table S1. A previous study showed that a hydrolysate of mixed flour from sheep leather and wool waste showed low molecular peptides mainly originating from keratin protein, along with an elevation in free amino acids (aa) such as Asn, Lys, Pro, and Gly (Gousterova et al. 2005).

Moreover, the DH of BSPH using GID was 14.03% ± 0.29%. It resembled the research by Liang et al. 2014, which indicates the value of DH after GID around 16.74% from collagen of *Nile tilapia* skin. Another study also showed hydrolysis of Alaska pollock skin collagen using enzymes simultaneously, such as alcalase, gastric, and two times of intestinal digestion, resulting in DH of 13.17%, 16.92%, and 26%, respectively (Sun et al. 2016).

Table 1 displays the concentrations of free aa in BSP and BSPH. The total concentration of free aa rose from 0.265 ± 0.012 to 1039.073 ± 40.233 mg aa/g after GID, which indicates that gastrointestinal proteases successfully hydrolyzed the BSP sample and generated short‐chain peptides and free aa. The linear regression data for every amino acid in free amino acid analysis were present in Table S6. The most abundant free aa of BSPH were Lys, Arg, Leu, and Tyr. The abundance of Arg might be caused by the cleavage specificity of the bromelain enzyme, which cleaves at benzyloxycarbonyl‐Arg‐Arg and basic aa such as Lysine (Tacias‐Pascacio et al. 2023; Azarkan et al. 2020). This enzymatic preference can lead to the release of free residues or peptides with terminal Arg or Lys. Moreover, a previous study by Zeng et al. (2022) found that Lys, Glu, Phe, Leu, and Arg are abundant amino acids in pork skin proteins after GID.

**TABLE 1:** Free amino acids profile of bromelain‐soluble protein (BSP), bromelain‐soluble protein hydrolysate (BSPH), and control of gastrointestinal digestion (GID).

mg aa/g	Sample		
	BSP	BSPH	Control GID
Asp	0.012 ± 0.002^c^	26.885 ± 0.861^a^	4.200 ± 0.437^b^
Glu	0.029 ± 0.009^c^	61.098 ± 1.754^a^	11.622 ± 0.869^b^
Ser	0.023 ± 0.002^c^	41.369 ± 1.392^a^	16.873 ± 0.484^b^
Asn	n.d.	35.233 ± 0.419^a^	14.972 ± 0.720^b^
Gly	0.014 ± 0.004^c^	27.315 ± 1.380^a^	10.356 ± 0.209^b^
Gln	n.d.	36.372 ± 0.164^a^	11.971 ± 0.414^b^
Taurine	n.d.	0.927 ± 0.142^a^	0.636 ± 0.014^b^
His	n.d.	27.643 ± 1.020^a^	11.259 ± 0.285^b^
Thr	0.015 ± 0.001^c^	46.916 ± 0.225^a^	15.346 ± 0.662^b^
Ala	0.037 ± 0.013^c^	44.886 ± 0.012^a^	24.9723 ± 0.228^b^
Carnosine	n.d.	2.620 ± 1.651^a^	n.d.
Arg	0.029 ± 0.008^c^	176.474 ± 2.428^a^	23.997 ± 0.197^b^
Pro	n.d.	12.296 ± 0.972^a^	7.959 ± 0.197^b^
Tyr	0.019 ± 0.013^c^	92.312 ± 10.0150^a^	70.604 ± 5.323^b^
Val	0.008 ± 0.001^c^	52.938 ± 4.567^a^	35.962 ± 2.242^b^
Met	n.d.	19.405 ± 1.700^a^	13.187 ± 0.723^b^
Cystine	n.d.	3.976 ± 0.663^a^	2.423 ± 0.027^b^
Ile	0.009 ± 0.001^c^	43.318 ± 4.504^a^	35.418 ± 3.148^b^
Leu	0.035 ± 0.009^c^	92.040 ± 9.779^a^	72.847 ± 6.559^b^
Hyl	n.d.	1.694 ± 0.240^a^	n.d.
Phe	0.012 ± 0.006^b^	55.267 ± 7.264^a^	50.265 ± 5.737^b^
Trp	n.d.	36.126 ± 4.913^a^	37.212 ± 4.528^a^
Orn	0.005 ± 0.002^c^	1.830 ± 0.0516^a^	0.314 ± 0.051^b^
Lys	0.017 ± 0.002^c^	100.132 ± 1.940^a^	15.537 ± 0.184^b^
Total	0.265 ± 0.012^c^	1039.073 ± 40.233^a^	482.594 ± 23.151^b^

*Note:* Superscript letters indicate significant differences between samples.

In general, the action of gastrointestinal enzymes promotes the breakdown of proteins into peptides and free aa, which may contribute to bioactivity. In this study, BSPH exhibited an ACE‐inhibitory activity of 48.99% ± 1.17%, which was significantly higher than that of BSP. In accordance with this, Alemán et al. (2013) indicated that GID enhanced the ACE‐inhibition capacity of squid skin collagen hydrolysates. Likewise, Guo et al. (2014) reported up to a 20‐fold increase in ACE‐inhibition activity of collagen derived from Alaska pollock skin following simulated GID. Collectively, these studies confirm that GID not only generates bioactive peptides but also enhances their inhibitory potential against ACE.

### 3.2 Peptide Fractionation and Bioactivity Profile

**FIGURE 1:**
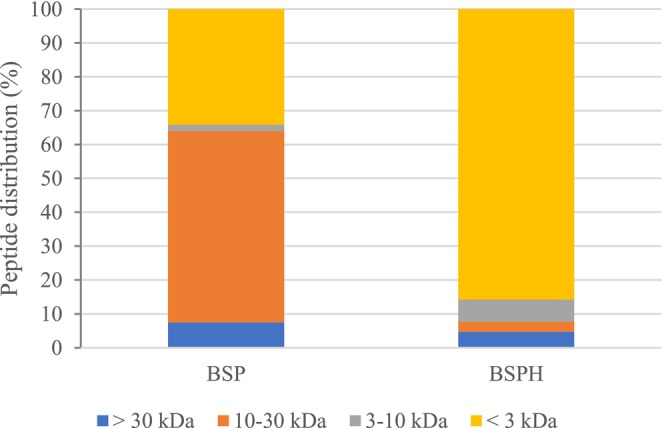
Molecular weight distribution of the peptides of bromelain‐soluble protein (BSP) and bromelain‐soluble protein hydrolysate (BSPH) isolated from sheepskin.

The BSP and BSPH samples were fractionated by ultrafiltration filter with specific size 30, 10, and 3 kDa to assess the molecular weight distribution of peptides, and the results are presented in Figure 1. BSP was mainly composed of oligopeptides and proteins between 10 and 30 kDa, representing 56.61%, whereas BSPH reached a percentage of 85.75% of peptides that are smaller than 3 kDa. The GID process generates small molecular weight peptides due to enzymatic cleavage (Correa et al. 2024), which might have great bioactivity, including ACE inhibition. Short‐chain peptides can adopt spatial conformations that enable them to fit in the 3D structure of the ACE enzyme, thereby restricting the accessibility of larger peptides (Abdelhedi and Nasri 2019). In addition, low‐molecular‐weight peptides are less susceptible to proteolytic degradation during gastrointestinal digestion and have a higher likelihood of crossing the intestinal membrane intact, ultimately reaching the bloodstream (Theysgeur et al. 2020; Abdelhedi and Nasri 2019).

**FIGURE 2:**
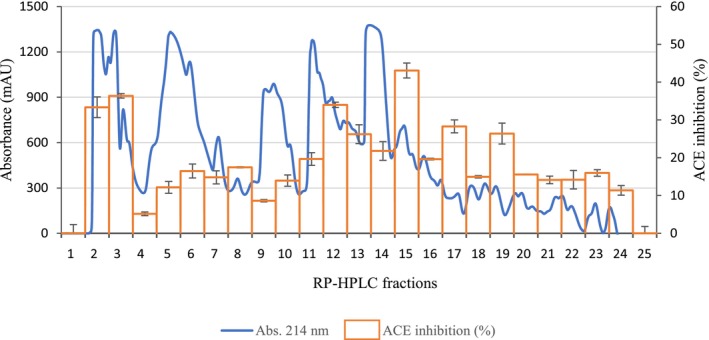
Reverse‐phase high‐performance liquid chromatography (RP‐HPLC) profile and ACE inhibition of the fraction < 3 kDa from bromelain‐soluble protein hydrolysate (BSPH) isolated from sheepskin.

The highest bioactivity is commonly attributed to small peptides (Gallego et al. 2018), so fractions < 3 kDa of BSP and BSPH were analyzed for ACE‐inhibitory capacity. No activity was found in BSP at 1 mg/mL while a value of 19.94% ± 2.59% was obtained for BSPH at the same concentration, which evidenced the generation of active peptides during GID. The fraction of BSPH < 3 kDa was further analyzed by RP‐HPLC as displayed in Figure 2. A total of 25 RP‐HPLC fractions were obtained and put through an ACE‐inhibitory activity test for ACE‐inhibitory activity (see Figure 2), with fractions 2, 3, 12, and 15 showing the highest values (33.35%, 36.37%, 33.99%, and 43.07%, respectively). Since the RP‐HPLC separation was based on peptide polarity, fractions 2 and 3 would be associated with peptides with hydrophilic behavior, whereas fractions 12 and 15 would contain peptides with more hydrophobic character. Therefore, the active fractions may be regarded as a mixture of polar (hydrophilic) and non‐polar (hydrophobic) peptides exhibiting ACE‐inhibitory potential. According to Mazloomi et al. (2020), hydrophobic peptides generally demonstrate stronger ACE‐inhibitory activity, whereas highly hydrophilic peptides tend to be less effective because of their limited ability to interact with the enzyme's active site. Nonetheless, peptides containing both hydrophilic and hydrophobic residues may also effectively bind to ACE, since the inhibitory activity is largely influenced by the proportion of hydrophobic aa within the sequence of the peptide.

### 3.3 Peptide Sequences Identification and In Silico Analysis

**FIGURE 3:**
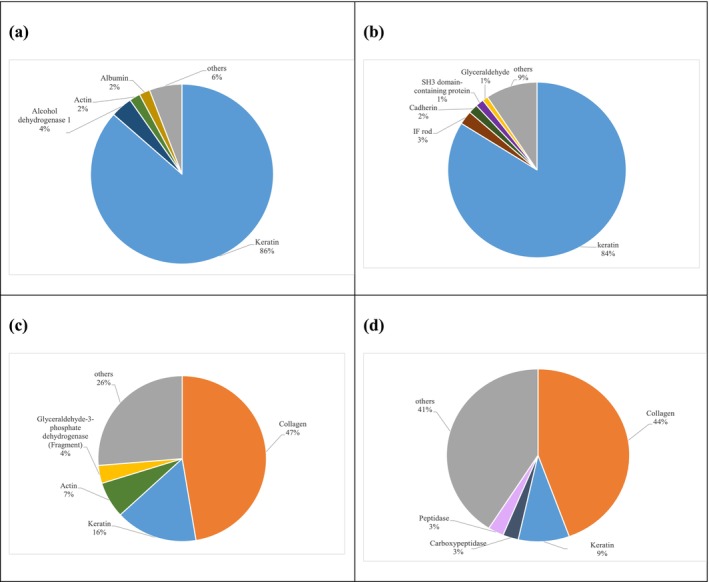
Distribution of the peptides identified by liquid chromatography–tandem mass spectrometry (LC–MS/MS) according to their parent protein in the (a) fraction 2, (b) fraction 3, (c) fraction 12, and (d) fraction 15 collected from reverse phase‐high performance liquid chromatography (RP‐HPLC).

The most active RP‐HPLC fractions (2, 3, 12, and 15) were analyzed by LC–MS/MS. Tables S2–S5 listed the identified peptides contained in each fraction as well as their protein of origin. Over 1560 peptide sequences were identified in the four fractions, with lengths between 7 and 30 amino acids, so that 56 peptides belonged to fraction 2, 1296 to fraction 3, 60 to fraction 12, and 148 to fraction 15. Figure 3 illustrates the distribution of the recognized peptides in accordance with the protein from which they originated, observing that most of the peptides were derived from connective tissue proteins such as keratin and collagen. In fact, fractions 2 and 3 were dominated by peptides derived from keratin, around 86% and 83%, respectively, whereas fractions 12 and 15 contained 47% and 44%, respectively, of collagen‐derived peptides. These proteins have hydrophobic character, but the release of peptides after hydrolysis would increase their hydrophilicity, so that they elute in the most polar fractions of RP‐HPLC. The remaining peptide sequences originated from other proteins, including actin, albumin, glyceraldehyde‐3‐phosphate dehydrogenase and alcohol dehydrogenase, SH3 protein, cadherin, peptidase, carboxypeptidase, and others. Banerjee and Shanthi (2012) reported that two peptides exhibit bioactivity with ACE‐inhibitory capacity in bovine Achilles tendon collagen hydrolysates generated by bacterial collagenase. The identified peptides scaled from 1.5 to 3.5 kDa and were characterized by a relatively high content of Ala and Asn, with Pro frequently located at the C‐terminal. Notably, both peptides maintained approximately 80% of their inhibitory activity following simulated gastrointestinal digestion.

In silico analysis serves as an efficient preliminary approach for screening bioactive peptides, offering both time and cost advantages before validation through in vitro assays (Xiao et al. 2022). In the current investigation, all recognized peptides were evaluated using the Peptide Ranker database to select sequences with the highest likelihood of exhibiting bioactivity. So, those peptides with PRR > 0.85, resulting in 79 sequences, are listed in Table 2. These peptides were further characterized using the BIOPEP‐UWM database to calculate A values, which scaled from 0.546 to 1.191, indicating a high presence of ACE‐inhibitory fragments in the identified peptides. AllerTOP and ToxinPred tools predicted that most of the sequences were non‐allergenic and non‐toxic. Peptide characteristics were also evaluated in silico (Table 2), obtaining hydrophobicity values that scaled between −0.2 and 0.26, steric hindrance and side bulk values from 0.39 to 0.68, hydrophathicity values from −0.79 to 0.79, and amphipathicity values of 0–0.37. The ability of the peptides to penetrate cells was also predicted, showing values that scaled from 0.044 to 0.269 (Table 2). Yin et al. (2022) stated that peptides showing low steric hindrance values would favor the binding capacity to the ACE active site, increasing inhibitory activity. Hydrophobic peptides are mainly related to high ACE inhibition activity, although other sequence characteristics, such as the occurrence of proline and aromatic amino acids, also determine peptide activity. On the other hand, Kalafatovic and Giralt (2017) stated that the amphipathicity of the peptides was related to their CPP value, so peptides with amphipathic structures can penetrate the cells more easily.

**TABLE 2:** In silico analysis results of the reverse phase‐high performance liquid chromatography (RP‐HPLC) fractions 2, 3, 12, and 15.

RP‐HPLC fraction	Peptide sequence	Parent protein	PRR^a^	A value^b^	Allergenicity	Toxicity	Hydrophobicity	Steric hindrance	Sidebulk	Hydropathicity	Amphipathicity	CPP^c^
F2	FSSCGGGGGSFGAGGGFGSR	Keratin	0.920	0.700	Not	Not	0.05	0.64	0.64	0.05	0.12	0.060
F3	GGGGGGGFNFG	Uncharacterized protein	0.851	0.909	Not	Not	0.17	0.69	0.69	−0.1	0	0.054
	GGGGGSFGAGGGFGS	Keratin	0.852	0.933	Not	Not	0.17	0.65	0.65	0.12	0	0.052
	SSSKGSLGGGFSSGGFSGGS	Keratin	0.857	0.600	Not	Not	−0.01	0.61	0.61	−0.21	0.18	0.050
	GPGGFGPGGYPGGIH	Keratin, type II	0.860	1.000	Not	Not	0.14	0.57	0.57	−0.35	0.1	0.057
	KGSLGGGFSSGGFSGGSF	Keratin, type I	0.861	0.722	Not	Not	0.07	0.63	0.63	0.06	0.2	0.053
	GGGFGGGFGGDGGLLSGNEK	Keratin, type I	0.869	0.850	Yes	Not	0.04	0.67	0.67	−0.32	0.25	0.079
	GPGVDKEPFNLF	Cadherin domain‐containing protein	0.871	0.611	Not	Not	−0.05	0.63	0.63	−0.4	0.41	0.087
	SSKGSLGGGFSSGGFSGG	Keratin, type I	0.871	0.611	Not	Not	0.02	0.62	0.62	−0.14	0.2	0.053
	GGGFGGGDGLL	Keratin, type I	0.871	1.000	Not	Not	0.19	0.66	0.66	0.37	0	0.082
	GGGFGGGFGGDGGLLS	Keratin, type I	0.872	0.938	Not	Not	0.18	0.66	0.66	0.31	0	0.065
	GGGFGGDGGLLSGNEKVTM	Keratin, type I	0.874	0.632	Not	Not	0.01	0.66	0.66	−0.14	0.26	0.101
	SLGGGFSSGGFSGGSF	Keratin, type I	0.877	0.688	Not	Not	0.14	0.63	0.63	0.34	0	0.045
	SLGGGFGGGSRGFGGA	Keratin, type o	0.878	0.938	Not	Not	0.07	0.64	0.64	0.09	0.15	0.085
	GGPGGFGPGGYPGGIH	Keratin, type II	0.880	1.000	Not	Not	0.14	0.58	0.58	−0.35	0.09	0.058
	SLGGGFGGGSRGF	Keratin, type I	0.881	0.846	Not	Not	0.05	0.65	0.65	0.04	0.19	0.074
	GSLGGGFSSGGFSGGSF	Keratin, type I	0.882	0.706	Not	Not	0.14	0.63	0.63	0.29	0	0.046
	APSADAPMFVM	Glyceraldehyde‐3‐phosphate dehydrogenase	0.882	0.546	Not	Not	0.12	0.59	0.59	0.79	0	0.091
	SSSKGSLGGGFSSGGFSGG	Keratin, type I	0.883	0.579	Not	Not	0.01	0.62	0.62	−0.17	0.19	0.051
	GGGGGSFGAGGGFGSR	Keratin, type II	0.883	0.875	Not	Not	0.05	0.65	0.65	−0.17	0.15	0.072
	GGGGYSSGGGGGGGYGSGGGGGYGSG	Keratin, type II	0.891	1.000	Not	Not	0.08	0.66	0.66	−0.57	0	0.051
	SKGSLGGGFSSGGFSGGSF	Keratin, type I	0.893	0.684	Not	Not	0.05	0.63	0.63	0.02	0.19	0.052
	GSGGGGFGGGGFGGGGYGGGY	Keratin, type II	0.896	1.191	Not	Not	0.17	0.68	0.68	−0.2	0	0.054
	GGGFGGGFGGDGGLLSGN	Keratin, type I	0.897	0.889	Not	Not	0.13	0.67	0.67	0.06	0	0.068
	SGGYGGLGGFGGGSFRGS	Keratin, type I	0.901	1.111	Not	Not	0.05	0.65	0.65	−0.16	0.14	0.064
	GGGGFGGGGFGGGGY	Keratin, type II	0.905	1.133	Not	Not	0.21	0.68	0.68	−0.03	0	0.055
	GGGYGGGGFGGGGFGGGGFGGGGIGGGGF	Keratin, type II	0.905	1.138	Not	Not	0.24	0.68	0.68	0.18	0	0.078
	GGGGFGGGGFGGGGIGGGGFGGF	Keratin, type II	0.907	1.087	Not	Not	0.26	0.68	0.68	0.37	0	0.061
	SSKGSLGGGFSSGGFSGGSF	Keratin, type I	0.910	0.650	Not	Not	0.04	0.62	0.62	−0.03	0.18	0.050
	GGSFGGGSFGGGSF	Keratin, type I	0.911	1.071	Not	Not	0.17	0.65	0.65	0.2	0	0.043
	GGGSGFSGGGFGGGGFGGGRF	Keratin, type II	0.914	1.000	Not	Not	0.11	0.67	0.67	−0.02	0.12	0.065

	SFGGGSGFSGGGFGGGGFGGGRFGGF	Keratin, type II	0.918	1.077	Not	Not	0.14	0.67	0.67	0.13	0.09	0.067
	GGGFGGDGGLL	Keratin, type I	0.920	0.909	Not	Not	0.19	0.66	0.66	0.37	0	0.082
	FSSCGGGGGSFGAGGGFGSR	Keratin, type II	0.920	0.700	Not	Not	0.05	0.64	0.64	0.05	0.12	0.060
	GGGSFGGGSFGGGSFG	Keratin, type I	0.920	1.063	Not	Not	0.17	0.66	0.66	0.12	0	0.044
	GGGSGFSGGGFGGGGFGGGRFGG	Keratin, type II	0.921	1.044	Not	Not	0.12	0.67	0.67	−0.06	0.11	0.071
	GGGSFGGGSFGGGSF	Keratin, type I	0.924	1.067	Not	Not	0.17	0.65	0.65	0.16	0	0.044
	GGGGGGFGGGGFGSR	Keratin, type II	0.924	0.933	Not	Not	0.06	0.67	0.67	−0.27	0.16	0.075
	GGGGFGGGGFGGGGFGGGGIGGGGF	Keratin, type II	0.929	1.080	Not	Not	0.25	0.68	0.68	0.31	0	0.071
	GSGGGGFGGGGFGGGGY	Keratin, type II	0.933	1.118	Not	Not	0.18	0.67	0.67	−0.1	0	0.052
	GSGGGGFGGGGF	Keratin, type II	0.939	1.000	Not	Not	0.2	0.67	0.67	0.1	0	0.048
	GGGSFGGGSFGGGSFGGG	Keratin, type I	0.941	1.111	Not	Not	0.17	0.66	0.66	0.07	0	0.046
	GGGFGGGGIGGGGF	Keratin, type II	0.942	1.000	Not	Not	0.27	0.68	0.68	0.41	0	0.056
	GGGFGGGFGGDGGLL	Keratin, type I	0.944	1.000	Not	Not	0.21	0.67	0.67	0.38	0	0.073
	GGPGGVGGLGGPGGFGPG	Keratin, type II	0.945	0.944	Not	Not	0.19	0.62	0.62	0.07	0	0.123
	SGGGFGGGGFGGGR	Keratin, type II	0.947	1.071	Not	Not	0.06	0.67	0.67	−0.26	0.18	0.073
	GGGGFGGGGFGGGGFGGGGIGGGGFGGF	Keratin, type II	0.947	1.107	Not	Not	0.26	0.68	0.68	0.35	0	0.072
	GGPGGVGGLGGPGGF	Keratin, type II	0.948	0.933	Not	Not	0.21	0.63	0.63	0.24	0	0.113
	GSGGGGFGGGGFGGG	Keratin, type II	0.949	1.067	Not	Not	0.19	0.67	0.67	0	0	0.055
	GGGGGGFGGGGF	Keratin, type II	0.955	1.000	Not	Not	0.23	0.68	0.68	0.13	0	0.057
	GGGGIGGGGFGGF	Keratin, type II	0.958	1.000	Not	Not	0.27	0.68	0.68	0.47	0	0.052
	GGGSGFSGGGFGGGGF	Keratin, type II	0.959	0.938	Not	Not	0.19	0.66	0.66	0.15	0	0.047
	GGFGSGGGGFGGGGF	Keratin, type II	0.960	1.000	Not	Not	0.22	0.67	0.67	0.21	0	0.049
	GGGGFGGGGIGGGGFGG	Keratin, type II	0.960	1.059	Not	Not	0.25	0.68	0.68	0.26	0	0.064
	GGGGFGGGGIGGGGF	Keratin, type II	0.960	1.000	Not	Not	0.26	0.68	0.68	0.35	0	0.059
	GGFGGGFGGGSGGGFGGGY	Keratin, type I	0.961	1.158	Not	Not	0.2	0.68	0.68	0.04	0	0.051
	SGGGFGGGGFGGGRFGG	Keratin, type II	0.962	1.118	Not	Not	0.1	0.67	0.67	−0.1	0.14	0.070
	GGGSGFSGGGFGGGGFGGGR	Keratin, type II	0.964	1.000	Not	Not	0.09	0.67	0.67	−0.17	0.12	0.068
	GGGSGFSGGGFGGGGFGGGRFGGF	Keratin, type II	0.965	1.042	Not	Not	0.14	0.67	0.67	0.06	0.1	0.070
	GGGSFGGGSFGGGSFGGGGF	Keratin, type I	0.969	1.100	Not	Not	0.19	0.66	0.66	0.18	0	0.047
	GGGSFGGGGFGGGGF	Keratin, type I	0.970	1.067	Not	Not	0.22	0.67	0.67	0.21	0	0.049
	SGGGFGGGGFGGGRF	Keratin, type II	0.972	1.067	Not	Not	0.09	0.67	0.67	−0.06	0.16	0.065
	GGGSFGGGSFGGGGFGGGGF	Keratin, type I	0.972	1.100	Not	Not	0.21	0.67	0.67	0.2	0	0.050
GGGGFGGGRFGGF	Keratin, type II	0.972	1.077	Not	Not	0.12	0.68	0.68	0.02	0.19	0.071

	GFGGGGFGGGRFGGF	Keratin, type II	0.973	1.133	Not	Not	0.15	0.69	0.69	0.18	0.16	0.067
	GGGGFGGGGFGGGGF	Keratin, type II	0.973	1.067	Not	Not	0.25	0.68	0.68	0.24	0	0.055
	GGGGFGGGGFGGGGIGGGGF	Keratin, type II	0.976	1.050	Not	Not	0.26	0.68	0.68	0.32	0	0.062
	GGGGFGGGGIGGGGFGGF	Keratin, type II	0.978	1.056	Not	Not	0.27	0.68	0.68	0.41	0	0.055
	SGGGFGGGGFGGGRFGGF	Keratin, type II	0.979	1.111	Not	Not	0.13	0.68	0.68	0.06	0.14	0.066
	GGGSFGGGGFGGGGFGGGF	Keratin, type I	0.981	1.105	Not	Not	0.23	0.68	0.68	0.25	0	0.053
GGGGFGGGGFGGGFGGGF	Keratin, type I	0.984	1.111	Not	Not	0.26	0.68	0.68	0.31	0	0.056
F12	**GPAGPAGPR**	Collagen type I alpha 2 chain	0.861	1.111	Yes	Not	−0.11	0.54	0.54	−0.77	0.27	0.267
	GPAGPAGPRG	Collagen type I alpha 2 chain	0.908	1.100	Yes	Not	−0.08	0.55	0.55	−0.73	0.25	0.247
	SSKGSLGGGFSSGGFSGGSF	Keratin, type I	0.910	0.650	Not	Not	0.04	0.62	0.62	−0.03	0.18	0.050
F15	AGPSGPNGPPGPA	Collagen type I alpha 2 chain	0.853	1.000	Not	Not	−0.01	0.53	0.53	−0.79	0	0.110
	PPGPAGPR	Collagen alpha‐1 (I) chain	0.865	1.000	Yes	Yes	−0.18	0.5	0.5	−1.24	0.31	0.269
	**QGPPGPAGPR**	Collagen alpha‐1 (I) chain	0.871	1.200	Yes	Yes	−0.2	0.54	0.54	−1.38	0.37	0.261
	GPSGPQGPSGPPGPK	Collagen alpha‐1 (I) chain	0.890	1.000	Not	Not	−0.13	0.53	0.53	−1.37	0.33	0.125
	GHPPPPPPPPE	Heterogeneous nuclear ribonucleoprotein L	0.895	1.3636	Not	Not	−0.13	0.39	0.39	−1.81	0.25	0.106

*Note:* Bold letter: the best sequences with the highest A and CPP values.

^a^ PRR, Peptide Ranker Ratio. Only the peptides with values > 0.85 are included in the list.

^b^ Frequency of ocurrence of ACE‐inhibitory fragments in the peptide sequence, according to the BIOPEP database.

^c^ CPP: Probability to be cell penetrating peptides.

Among all results of in silico analysis (Table 2), GPAGPAGPR and QGPPGPAGPR peptides are promising candidates to be ACE inhibitory peptides, with A values > 1.11 and CPP values > 0.26 (Table 2). These selected peptides were recognized in the RP‐HPLC fractions 12 and 15, respectively, and both were derived from collagen protein. They contained Pro residues close to both C‐ and N‐terminal and showed low hydrophobicity values (−0.11 and −0.2, respectively). The occurrence of hydrophobic aa residues, particularly Leu, Pro, Phe, Val, Ile, or Ala at the C‐terminal region has been reported as a key determinant for the competitive inhibition of ACE. In addition, structural characteristics such as aliphatic aa at the N‐terminus have been shown to strengthen the inhibition capacity of peptides (Yin et al. 2022). Senadheera et al. (2022) identified promising ACE‐inhibitory peptides of sea cucumber by‐products hydrolysates that had a large range of hydrophobicity and hydrophilicity values, spanning from −0.05 to 0.43 and −0.9 to 0.25, respectively.

Thus, the diverse physicochemical properties of short‐chain peptides suggest their potential to exert functional activity across various food systems, including aqueous, lipid‐based, and emulsion matrices. On the other hand, despite GPAGPAGPR and QGPPGPAGPR not showing very high CPP values, these peptides contain active fragments that could exert their function. In this sense, Table 3 describes the sequences contained in both peptides previously documented as ACE‐inhibitory peptides according to the database maintained by BIOPEP‐UWM. Several fragments of these two peptides contain Pro residues, which have an important role for ACE inhibition activity. This is owing to the fact that proline has a considerable affinity for the aa residues that are present at the enzyme's active site (Cheung et al. 1980). Among the potential fragments, PR showed the lowest IC_50_ value (4.1 μM), indicating a potent ACE inhibitory activity, whereas PG presented the highest value (17,000 μM). However, the correlation between the sequence length and the efficacy of inhibiting ACE was still poorly characterized, so a peptide with a longer sequence may not have lower activity compared with di‐ or tri‐peptides. Many studies have reported potent ACE inhibitor peptides with longer sequences, such as FMPGVPGPIQR, whose IC_50_ value reached 11.08 μM (Wang et al. 2020), or IVGRPRHQG that showed an IC_50_ value of 6.2 μM (Yokoyama et al. 1992). Therefore, further investigation is required to validate the ACE inhibitory capacity of the two selected peptides using in vivo and in vitro confirmation analyses. Comprehensive validations would provide robust scientific evidence supporting the incorporation of these selected peptide applications in functional food formulations as well as medical applications. The detailed study such as bioassay, inhibition mechanism, oligopeptide structure related to bioactivity are needed before promoting their future application and commercialization as natural bioactive ingredients.

**TABLE 3:** Potential sequences of the selected peptides showing ACE‐inhibitory activity according to previous studies.

Peptide	Sequence	IC_50_ (uM)	Location	References
GPAGPAGPR	GPA	405	[1–3], [4–6]	Meisel et al. (2006)
	PR	4.1	[8–9]	Saito et al. (1994)
	GP	252.63	[1–2], [4–5], [7–8]	Byun and Kim (2002)
	AG	2500	[3–4], [6–7]	Cheung et al. (1980)
	AGP	560	[3–5], [6–8]	Ichimura et al. (2003)
QGPPGPAGPR	GPA	405	[5–7]	Meisel et al. (2006)
	PR	4.1	[9–10]	Saito et al. (1994)
	GP	252.63	[2–3], [5–6], [8–9]	Byun and Kim (2002)
	AG	2500	[7–8]	Cheung et al. (1980)
	QG	7400	[1–2]	Cheung et al. (1980)
	PG	17,000	[4–5]	Cheung et al. (1980)
	GPP	23.1	[2–4]	Murray and FitzGerald (2007)
	PP	n.d.	[3–4]	Van Platerink et al. (2008)
	AGP	560	[7–9]	Ichimura et al. (2003)
	QGP	149.5	[1–3]	O'Keeffe et al. (2017)

## 4 Conclusions

The present study revealed that main sheepskin proteins extracted by bromelain corresponded to keratin and collagen. The gastrointestinal digestion (GID) effectively hydrolyzed the BSP, yielding a large increase in free amino acids and low molecular weight peptides. The low molecular fraction < 3 kDa exhibited the highest possible capacity to decrease ACE activity. The peptide sequences identification analyses by MS/MS recognized a total of 1560 peptide sequences in the most active reverse‐phase HPLC fractions, and in silico analyses revealed two novel peptides, GPAGPAGPR and QGPPGPAGPR, as promising ACE inhibitors. Nevertheless, additional investigations must be conducted to validate the bioactivity of these peptides, particularly through in vitro confirmation and in vivo evaluation.

## Author Contributions

**Dita Prameswari Trenggono Putri:** writing – original draft, formal analysis, investigation, data curation. **Marta Gallego:** writing – review and editing, methodology, data curation. **Mohammad Zainal Abidin:** writing – review and editing. **Nanung Agus Fitriyanto:** writing – review and editing. **Leticia Mora:** writing – review and editing, methodology, conceptualization, data curation, supervision. **Fidel Toldrá:** writing – review and editing, methodology, supervision, conceptualization. **Yuny Erwanto:** conceptualization, funding acquisition, writing – review and editing, supervision, validation.

## Ethics Statement

There is no need for ethical clearance due to using sheepskin from the local market.

## Conflicts of Interest

The authors declare no conflicts of interest.

## Supporting information


**Figure S1:** SDS‐PAGE pattern of bromelain‐soluble protein (BSP) and bromelain‐soluble protein hydrolysate (BSPH) isolated from sheepskin. The red rectangle corresponds to the nine protein bands that were selected for LC–MS/MS analysis.

**Table S1:** Protein identification of SDS‐PAGE bands in the bromelain‐soluble sheepskin protein extract.
**Table S2:** Peptide sequences identified by LC–MS/MS in the RP‐HPLC fraction 2 of the bromelain‐soluble sheepskin protein extract after gastrointestinal digestion.
**Table S3:** Peptide sequences identified by LC–MS/MS in the RP‐HPLC fraction 3 of the bromelain‐soluble sheepskin protein extract after gastrointestinal digestion.
**Table S4:** Peptide sequences identified by LC–MS/MS in the RP‐HPLC fraction 12 of the bromelain‐soluble sheepskin protein extract after gastrointestinal digestion.
**Table S5:** Peptide sequences identified by LC–MS/MS in the RP‐HPLC fraction 15 of the bromelain‐soluble sheepskin protein extract after gastrointestinal digestion.
**Table S6:** Linear regression for every amino acid in free amino acid analysis.

## Data Availability

The data that support the findings of this study are available on request from the corresponding author. The data are not publicly available due to privacy or ethical restrictions.

## References

[fsn371396-bib-0001] Abdelhedi, O. , and M. Nasri . 2019. “Basic and Recent Advances in Marine Antihypertensive Peptides: Production, Structure‐Activity Relationship and Bioavailability.” Trends in Food Science & Technology 88: 543–557. 10.1016/j.tifs.2019.04.002.

[fsn371396-bib-0002] Adler‐Nissen, J. 1986. Enzymic Hydrolysis of Food Proteins. Elsevier Applied Science Publishers Ltd.

[fsn371396-bib-0003] Alemán, A. , M. C. Gómez‐Guillén , and P. Montero . 2013. “Identification of Ace‐Inhibitory Peptides From Squid Skin Collagen After In Vitro Gastrointestinal Digestion.” Food Research International 54: 790–795. 10.1016/j.foodres.2013.08.027.

[fsn371396-bib-0004] AOAC . 1992. “Official Method 992.15.” In Official Methods of Analysis of the AOAC International. AOAC International.

[fsn371396-bib-0005] Azarkan, M. , E. Maquoi , F. Delbrassine , et al. 2020. “Structures of the Free and Inhibitors‐Bound Forms of Bromelain and Ananain From *Ananas Comosus* Stem and *in Vitro* Study of Their Cytotoxicity.” Scientific Reports 10, no. 1: 19570. 10.1038/s41598-020-76172-5. 33177555 PMC7658999

[fsn371396-bib-0006] Banerjee, P. , and C. Shanthi . 2012. “Isolation of Novel Bioactive Regions From Bovine Achilles Tendon Collagen Having Angiotensin I‐Converting Enzyme‐Inhibitory Properties.” Process Biochemistry 47: 2335–2346. 10.1016/j.procbio.2012.09.012.

[fsn371396-bib-0007] Brodkorb, A. , L. Egger , M. Alminger , et al. 2019. “INFOGEST Static *in Vitro* Simulation of Gastrointestinal Food Digestion.” Nature Protocols 14, no. 4: 991–1014. 10.1038/s41596-018-0119-1. 30886367

[fsn371396-bib-0008] Byun, H.‐G. , and S.‐K. Kim . 2002. “Structure and Activity of Angiotensin I Converting Enzyme Inhibitory Peptides Derived From Alaskan Pollack Skin.” Journal of Biochemistry and Molecular Biology 35: 239–243. 10.5483/BMBRep.2002.35.2.239. 12297036

[fsn371396-bib-0009] Cheung, H. S. , F. L. Wang , M. A. Ondetti , E. F. Sabo , and D. W. Cushman . 1980. “Binding of Peptide Substrates and Inhibitors of Angiotensin‐Converting Enzyme. Importance of the COOH‐Terminal Dipeptide Sequence.” Journal of Biological Chemistry 255: 401–407. 10.1016/S0021-9258(19)86187-2. 6243277

[fsn371396-bib-0010] Correa, J. L. , J. E. Zapata , and B. Hernández‐Ledesma . 2024. “Impact of Alcalase Hydrolysis and Simulated Gastrointestinal Digestion on the Release of Bioactive Peptides From Erythrina Edulis (Chachafruto) Proteins.” International Journal of Molecular Sciences 25: 9290. 10.3390/ijms25179290. 39273238 PMC11394852

[fsn371396-bib-0011] Devita, L. , M. Nurilmala , H. N. Lioe , and M. T. Suhartono . 2021. “Chemical and Antioxidant Characteristics of Skin‐Derived Collagen Obtained by Acid‐Enzymatic Hydrolysis of Bigeye Tuna (*Thunnus obesus*).” Marine Drugs 19: 222. 10.3390/md19040222. 33923409 PMC8072911

[fsn371396-bib-0048] Fan, Y. , Z. Yu , W. Zhao , et al. 2020. “Identification and Molecular Mechanism of Angiotensin‐Converting Enzyme Inhibitory Peptides From *Larimichthys crocea* titin.” Food Science and Human Wellness 9, no. 3: 257–263. 10.1016/j.fshw.2020.04.001.

[fsn371396-bib-0012] Gallego, M. , L. Mora , E. Escudero , and F. Toldrá . 2018. “Bioactive Peptides and Free Amino Acids Profiles in Different Types of European Dry‐Fermented Sausages.” International Journal of Food Microbiology 276: 71–78. 10.1016/j.ijfoodmicro.2018.04.009. 29674143

[fsn371396-bib-0013] Gousterova, A. , D. Braikova , I. Goshev , et al. 2005. “Degradation of Keratin and Collagen Containing Wastes by Newly Isolated Thermoactinomycetes or by Alkaline Hydrolysis.” Letters in Applied Microbiology 40: 335–340. 10.1111/j.1472-765X.2005.01692.x. 15836735

[fsn371396-bib-0014] Guo, L. , P. A. Harnedy , L. Zhang , et al. 2014. “In Vitro Assessment of the Multifunctional Bioactive Potential of Alaska Pollock Skin Collagen Following Simulated Gastrointestinal Digestion.” Journal of the Science of Food and Agriculture 95, no. 7: 1514–1520. 10.1002/jsfa.6854. 25082083

[fsn371396-bib-0015] Hakim, T. R. , A. Pratiwi , J. Jamhari , et al. 2021. “Extraction of Collagen From the Skin of Kacang Goat and Production of Its Hydrolysate as an Inhibitor of Angiotensin Converting Enzyme.” Tropical Animal Science Journal 44: 222–228. 10.5398/tasj.2021.44.2.222.

[fsn371396-bib-0049] He, B. , Y. Lian , H. Xue , et al. 2024. “DPP‐IV Inhibitory Peptide Against In Vitro Gastrointestinal Digestion Derived from Goat's Milk Protein and its Activity Enhancement via Amino Acid Substitution.” Food 13, no. 17: 2721. 10.3390/foods13172721. PMC11395612 39272487

[fsn371396-bib-0016] Hou, Y. , B. Chitrakar , K. Mao , et al. 2023. “Bioactivity of Collagen Peptides Derived From Commercial Animals: In Silico Investigation.” LWT 187: 115381. 10.1016/j.lwt.2023.115381.

[fsn371396-bib-0017] Ichimura, T. , J. Hu , D. Q. Aita , and S. Maruyama . 2003. “Angiotensin I‐Converting Enzyme Inhibitory Activity and Insulin Secretion Stimulative Activity of Fermented Fish Sauce.” Journal of Bioscience and Bioengineering 96, no. 5: 496–499. 10.1016/S1389-1723(03)70138-8. 16233562

[fsn371396-bib-0018] Kalafatovic, D. , and E. Giralt . 2017. “Cell‐Penetrating Peptides: Design Strategies Beyond Primary Structure and Amphipathicity.” Molecules 22: 1929. 10.3390/molecules22111929. 29117144 PMC6150340

[fsn371396-bib-0019] Kotowicz, Z. , A. Pich‐Czekierda , P. Proszowska , et al. 2024. “Advantages of Oral Collagen Supplementation. Review of the Literature.” Journal of Education, Health and Sport 70: 50183. 10.12775/JEHS.2024.70.50183.

[fsn371396-bib-0020] Laemmli, U. K. 1970. “Cleavage of Structural Proteins During the Assembly of the Head of Bacteriophage T4.” Nature 227: 680–685. 10.1038/227680a0. 5432063

[fsn371396-bib-0021] Liang, Q. , L. Wang , Y. He , Z. Wang , J. Xu , and H. Ma . 2014. “Hydrolysis Kinetics and Antioxidant Activity of Collagen Under Simulated Gastrointestinal Digestion.” Journal of Functional Foods 11: 493–499. 10.1016/j.jff.2014.08.004.

[fsn371396-bib-0022] López‐Pedrouso, M. , P. Borrajo , M. Pateiro , J. M. Lorenzo , and D. Franco . 2020. “Antioxidant Activity and Peptidomic Analysis of Porcine Liver Hydrolysates Using Alcalase, Bromelain, Flavourzyme and Papain Enzymes.” Food Research International 137: 109389. 10.1016/j.foodres.2020.109389. 33233091

[fsn371396-bib-0023] Luasiri, P. , P. Sangsawad , J. Pongsetkul , et al. 2024. “Exploration of Nutritional and Bioactive Peptide Properties in Goat Meat From Various Primal Cuts During In Vitro Gastrointestinal Digestion and Absorption.” Animal Bioscience 37, no. 6: 1096–1109. 10.5713/ab.23.0352. 38575133 PMC11065958

[fsn371396-bib-0024] Matinong, A. M. E. , Y. Chisti , K. L. Pickering , and R. G. Haverkamp . 2022. “Collagen Extraction From Animal Skin.” Biology 11, no. 6: 905. 10.3390/biology11060905. 35741426 PMC9219788

[fsn371396-bib-0025] Mazloomi, S. N. , L. Mora , M.‐C. Aristoy , et al. 2020. “Impact of Simulated Gastrointestinal Digestion on the Biological Activity of an Alcalase Hydrolysate of Orange Seed (Siavaraze, *Citrus sinensis*) By‐Products.” Food 9: 1217. 10.3390/foods9091217. PMC7554958 32887246

[fsn371396-bib-0026] Meisel, H. , D. J. Walsh , B. Murray , and R. J. FitzGerald . 2006. “ACE Inhibitory Peptides.” In Nutraceutical Proteins and Peptides in Health and Disease, edited by Y. Mine and F. Shahidi , 269–315. CRC Press Taylor & Francis Group.

[fsn371396-bib-0027] Minekus, M. , M. Alminger , P. Alvito , et al. 2014. “A Standardised Static In Vitro Digestion Method Suitable for Food—An International Consensus.” Food & Function 5, no. 6: 1113–1124. 10.1039/C3FO60702J. 24803111

[fsn371396-bib-0028] Munmun, S. A. , T. U. Rashid , and M. M. Rahman . 2024. “Optimization of Enhanced Collagen Extraction From Tannery Rawhide Trimming Waste Using Pineapple Peel‐Derived Bromelain Enzyme Through Response Surface Methodology.” Journal of Cleaner Production 438: 140774. 10.1016/j.jclepro.2024.140774.

[fsn371396-bib-0029] Murray, B. , and R. FitzGerald . 2007. “Angiotensin Converting Enzyme Inhibitory Peptides Derived From Food Proteins: Biochemistry, Bioactivity and Production.” Current Pharmaceutical Design 13: 773–791. 10.2174/138161207780363068. 17430180

[fsn371396-bib-0030] Nielsen, P. M. , D. Petersen , and C. Dambmann . 2001. “Improved Method for Determining Food Protein Degree of Hydrolysis.” Journal of Food Science 66: 642–646. 10.1111/j.1365-2621.2001.tb04614.x.

[fsn371396-bib-0031] O'Keeffe, M. B. , R. Norris , M. A. Alashi , R. E. Aluko , and R. J. FitzGerald . 2017. “Peptide Identification in a Porcine Gelatin Prolyl Endoproteinase Hydrolysate With Angiotensin Converting Enzyme (ACE) Inhibitory and Hypotensive Activity.” Journal of Functional Foods 34: 77–88. 10.1016/j.jff.2017.04.018.

[fsn371396-bib-0032] Ozturk‐Kerimoglu, B. , A. Heres , L. Mora , and F. Toldrá . 2023. “Antioxidant Peptides Generated From Chicken Feet Protein Hydrolysates.” Journal of the Science of Food and Agriculture 103: 7207–7217. 10.1002/jsfa.12802. 37347843

[fsn371396-bib-0033] Saito, Y. , K. Wanezaki (Nakamura) , A. Kawato , and S. Imayasu . 1994. “Structure and Activity of Angiotensin I Converting Enzyme Inhibitory Peptides From Sake and Sake Lees.” Bioscience, Biotechnology, and Biochemistry 58: 1767–1771. 10.1271/bbb.58.1767. 7765503

[fsn371396-bib-0034] Senadheera, T. R. L. , A. Hossain , D. Dave , and F. Shahidi . 2022. “In Silico Analysis of Bioactive Peptides Produced From Underutilized Sea Cucumber By‐Products—A Bioinformatics Approach.” Marine Drugs 20: 610. 10.3390/md20100610. 36286434 PMC9605078

[fsn371396-bib-0035] Sentandreu, M. Á. , and F. Toldrá . 2006. “A Rapid, Simple and Sensitive Fluorescence Method for the Assay of Angiotensin‐I Converting Enzyme.” Food Chemistry 97, no. 3: 546–554. 10.1016/j.foodchem.2005.06.006.

[fsn371396-bib-0036] Shevchenko, A. , O. N. Jensen , A. V. Podtelejnikov , et al. 1996. “Linking Genome and Proteome by Mass Spectrometry: Large‐Scale Identification of Yeast Proteins From Two Dimensional Gels.” Proceedings of the National Academy of Sciences of the United States of America 93, no. 25: 14440–14445. 10.1073/pnas.93.25.14440. 8962070 PMC26151

[fsn371396-bib-0037] Sun, L. , W. Chang , Q. Ma , and Y. Zhuang . 2016. “Purification of Antioxidant Peptides by High Resolution Mass Spectrometry From Simulated Gastrointestinal Digestion Hydrolysates of Alaska Pollock (*Theragra chalcogramma*) Skin Collagen.” Marine Drugs 14: 186. 10.3390/md14100186. 27763502 PMC5082334

[fsn371396-bib-0038] Tacias‐Pascacio, V. G. , D. Castañeda‐Valbuena , O. Tavano , Á. B. Murcia , B. Torrestina‐Sánchez , and R. Fernandez‐Lafuente . 2023. “Peptides With Biological and Technofunctional Properties Produced by Bromelain Hydrolysis of Proteins From Different Sources: A Review.” International Journal of Biological Macromolecules 253: 127244. 10.1016/j.ijbiomac.2023.127244. 37806416

[fsn371396-bib-0039] Theysgeur, S. , B. Cudennec , B. Deracinois , et al. 2020. “New Bioactive Peptides Identified From a Tilapia Byproduct Hydrolysate Exerting Effects on DPP‐IV Activity and Intestinal Hormones Regulation After Canine Gastrointestinal Simulated Digestion.” Molecules 26: 136. 10.3390/molecules26010136. 33396793 PMC7796187

[fsn371396-bib-0040] Van Platerink, C. J. , H.‐G. M. Janssen , and J. Haverkamp . 2008. “Application of At‐Line Two‐Dimensional Liquid Chromatography–Mass Spectrometry for Identification of Small Hydrophilic Angiotensin I‐Inhibiting Peptides in Milk Hydrolysates.” Analytical and Bioanalytical Chemistry 391: 299–307. 10.1007/s00216-008-1990-3. 18392815 PMC2324126

[fsn371396-bib-0041] Wang, R. , X. Lu , Q. Sun , J. Gao , L. Ma , and J. Huang . 2020. “Novel Ace Inhibitory Peptides Derived From Simulated Gastrointestinal Digestion In Vitro of Sesame (*Sesamum indicum L*.) Protein and Molecular Docking Study.” International Journal of Molecular Sciences 21: 1059. 10.3390/ijms21031059. 32033479 PMC7037947

[fsn371396-bib-0042] World Health Organization (WHO) . 2023. “Hypertension.” https://www.who.int/news‐room/fact‐sheets/detail/hypertension. Accessed October 12, 2024.

[fsn371396-bib-0043] Xiao, C. , F. Toldrá , M. Zhao , et al. 2022. “In Vitro and In Silico Analysis of Potential Antioxidant Peptides Obtained From Chicken Hydrolysate Produced Using Alcalase.” Food Research International 157: 111253. 10.1016/j.foodres.2022.111253. 35761565

[fsn371396-bib-0044] Yang, G. , S. Qin , and W. Li . 2021. “Purification and Characterization of a Novel Angiotensin I‐Converting Enzyme‐Inhibitory Peptide Derived From Alaska Pollack Skins.” Journal of Food Science 86: 2457–2467. 10.1111/1750-3841.15754. 34056723

[fsn371396-bib-0045] Yin, Z. , R. Yan , Y. Jiang , et al. 2022. “Identification of Peptides in Qingke Baijiu and Evaluation of Its Angiotensin Converting Enzyme (ACE) Inhibitory Activity and Stability.” Food Chemistry 395: 133551. 10.1016/j.foodchem.2022.133551. 35802984

[fsn371396-bib-0046] Yokoyama, K. , H. Chiba , and M. Yoshikawa . 1992. “Peptide Inhibitors for Angiotensin I‐Converting Enzyme From Thermolysin Digest of Dried Bonito.” Bioscience, Biotechnology, and Biochemistry 56: 1541–1545. 10.1271/bbb.56.1541. 1369054

[fsn371396-bib-0047] Zeng, X. , B. Lv , K. Zhang , et al. 2022. “Digestion Profiles of Protein in Edible Pork By‐Products.” Food 11: 3191. 10.3390/foods11203191. PMC9602065 37430940

