# Supplementary material for: Characterization of Bromelain‐Soluble Sheepskin (Ovis aries) Proteins and Effect of In Vitro Gastrointestinal Digestion on Angiotensin Converting Enzyme Inhibition Activity
Source: Food Sci Nutr. 2026 Jan 28;14(2):e71396. doi: 10.1002/fsn3.71396 (PMC12848775; doi:10.1002/fsn3.71396)
Supplement: Supplementary file 1 — Figure S1: SDS‐PAGE pattern of bromelain‐soluble protein (BSP) and bromelain‐soluble protein hydrolysate (BSPH) isolated from sheepskin. The red rectangle corresponds to the nine protein bands that were selected for LC–MS/MS analysis. [file FSN3-14-e71396-s001.docx]

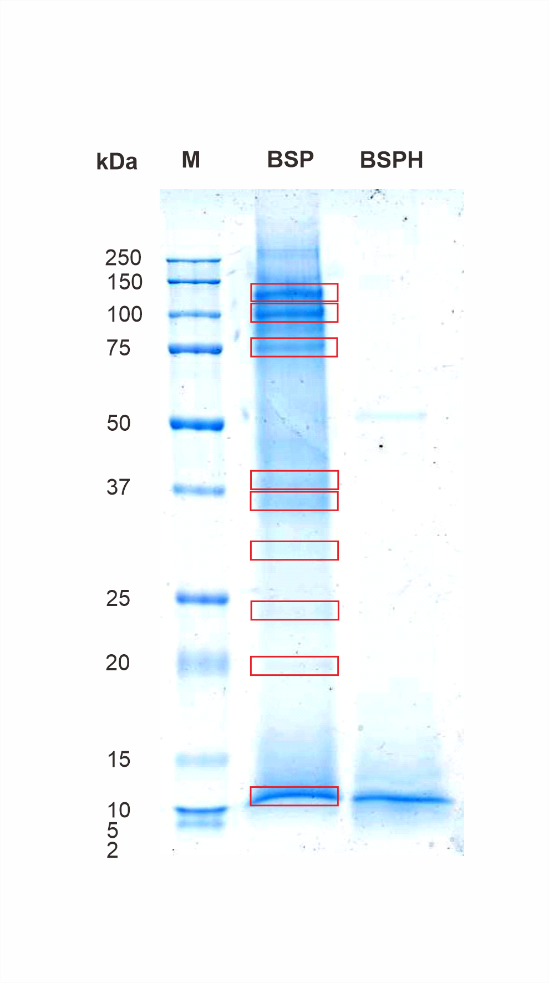


**Supplementary Figure**. SDS-PAGE pattern of bromelain-soluble protein (BSP) and bromelain-soluble protein hydrolysate (BSPH) isolated from sheepskin. The red rectangle corresponds to the nine protein bands that were selected for LC-MS/MS analysis.
